# Quantity–Quality Trade-Off and Early Childhood Development in Rural Family: Evidence from China’s Guizhou Province

**DOI:** 10.3390/ijerph16071307

**Published:** 2019-04-11

**Authors:** Jingdong Zhong, Jingjing Gao, Chengfang Liu, Jie Huang, Renfu Luo

**Affiliations:** 1 School of Economics, Peking University, Beijing 100871, China; jdzhong@pku.edu.cn; 2 China Center for Agricultural Policy, School of Advanced Agricultural Sciences, Peking University, Beijing 100871, China; gaojj@pku.edu.cn (J.G.); cfliu.ccap@pku.edu.cn (C.L.); 3 School of Economics, Jiangxi University of Finance and Economics, Nanchang 330013, China; hjiecn@gmail.com; 4 School of Economics and Management, Jiangxi Agricultural University, Nanchang 330045, China

**Keywords:** early childhood development, Quantity–Quality trade-off, intrahousehold resource allocation, home environment, parental warmth

## Abstract

This paper empirically investigates the causal effect of having siblings on the cognitive, language, motor, and social-emotional skills of infants under the age of 2 in rural families in Guizhou Province in China. The results are based on data from a survey conducted in 2017. To effectively relieve the endogeneity induced by selection bias, we applied the matching-smoothing (MS) method to evaluate the effects of having siblings. The results show that, first, having siblings produces significant negative impacts on an infant’s cognitive, language, and social-emotional skills; second, intrahousehold resource allocation is the mechanism behind the Quantity–Quality (Q–Q) trade-off, and it exerts its effects through two key identified channels—the home environment and parental warmth. By spreading the parents’ investment among siblings in terms of both the home environment and parental warmth, having siblings hinders infants’ early development. Our findings provide new evidence for the relation between the Q–Q trade-off and early childhood development in rural families in western China.

## 1. Introduction

Since the pioneering work of Gary Becker and his collaborators [1,2,3], the issue of the Quantity–Quality (Q–Q) trade-off has generated discussions worldwide [1,2,3,4,5,6,7,8,9,10]. Some work has documented that increasing the quantity of children in a family produces an adverse impact on the long-term welfare of those children; examples of affected outcomes include education attainment and labor-market outcomes [4,5].

However, this empirical relationship has been challenged by many studies, which have cited omitted-variable bias as the principal problem when establishing such causal inference between the quantity of children and its long-term outcomes on human capital and income [6,7,8,9,10]. Omitted-variable bias occurs when the relevant variables are not included in the econometric model, so it violates the assumption that the error term is uncorrelated with the regressors and causes bias in the ordinary least squares (OLS) estimator. For example, unobserved family heterogeneity, such as parents’ capacity [11], usually affects parents’ fertility choices and children’s development. Thus, applying the OLS estimator without accounting for parents’ capacity will result in omitted-variable bias.

The instrumental variable (IV) approach is commonly used to address this problem. A valid IV is correlated with the endogenous independent variable (quantity of children) but uncorrelated with the error term (exclusion restriction). The IV affects the dependent variable (children’s outcome) only through its effect on the endogenous independent variable (quantity of children). In the first stage of the approach, the endogenous variable (quantity of children) is regressed on both exogenous covariates and the excluded IV. In the second stage, the regression is estimated as the OLS except that the endogenous variable is replaced with its predicted value obtained from the first stage. This IV estimator is unbiased and consistent. However, the studies conducted in this way have obtained different results. The first work using twin birth as the IV for family size found that children in India from larger families had lower average schooling [12]. Similar results were found in China, too [13,14,15]. In the USA, twin birth [16] and the gender composition of siblings [17] were applied as IVs in separate studies, and both results indicated that children’s private school attendance was negatively correlated with the family size. In terms of magnitude, those studies identified non-trivial effect sizes (ranging from 0.2 to 0.4) of family size on children’s outcomes. In contrast to these results, other studies that adopted an IV have found little evidence of the Q–Q trade-off. Twins were applied as an IV in a large sample from Norway, and no negative effect of family size was found [6]. Multiple IV strategies were used in an Israeli Census sample, and no trade-off was found in this study either [10]. Similarly, no significant trade-off was found in a dataset from the USA [8].

Apart from twins and sibling gender, the exogenous policy shock is also used to study the natural variation in family size. In the Chinese context, some studies have applied the famous One-Child Policy (OCP) to identify the trade-off, but the findings of these studies have also been inconsistent [18]. The relaxation of the policy was used as an exogenous shock, and the results showed that an additional child promoted the first-born child’s school attendance, which does not support the existence of a trade-off [7]. The local OCP enforcement intensity was adopted as the exogenous variation, and the results revealed that the OCP contributed to the development of human capital in China [19]. A regression discontinuity design based on the OCP found a positive effect of birth control on children’s education [20]. The latter two studies both presented evidence to support the occurrence of a trade-off.

There are two reasons for the divergent findings on the Q–Q tradeoff for studies using a twin birth IV approach in the literature. One reason is the methodological differences between the studies. The IV estimator is sensitive to the external validity and variance of IV, resulting in conditions that have been hardly satisfied in almost all cases [21]. For example, in the male sample from the study in Norway, the use of twin birth as the IV led to IV estimates that revealed a negative effect of family size on children’s IQ. However, when employing the gender composition of siblings as the IV, there was no negative effect found [22]. The other reason is the contextual differences, such as the social economic status of families in the samples, between the studies. For example, as reviewed above, a negative correlation between family size and schooling was observed in the context of India [12] and China [13,14,15], whereas no significant relation was found in Norway [6] or Israel [10]. In developing countries such as India and China, the household budget constraint is much tighter, and a twin birth causes the parents to reduce the investment in each child, so the Q–Q trade-off holds. By contrast, in developed countries such as Norway and Israel, the families are richer on average, and they can ensure the same investment in children by intrahousehold reallocation, so a twin birth does not necessarily threaten the quality of children, i.e., the Q–Q trade-off does not hold.

Children’s long-term outcomes, the focus of most existing studies, are influenced by multi-stage investments spanning across their life cycle [23]. The effects of early investments are affected by later investments, too. As the later investments include many unobserved factors that originate from school and society during children’s growth [24], it is much more difficult to evaluate the real long-term effect of family size. For example, adolescence is a challenging transitional period for children. A systematic review found a strong linkage between adverse life events and suicidal behavior in young people [25]. Here, the adverse life events are the later negative investments. Hence, it is wiser to identify the real causal effects of family size on children’s short-term or early outcomes that are only influenced by early investments. Previous studies have also assessed long- versus short-term consequences in a way that might explain the contradictory findings. For example, twin birth was also adopted as an IV in a sample from Sweden, and the results revealed no impact of family size on children’s long-term outcomes, but they did show a significant negative impact on those children’s grades in compulsory and secondary school, i.e., their short-term performances [9].

In this paper, we analyze the direct impact of having siblings on early childhood development rather than children’s long-term welfares. For one thing, early childhood plays a vital role in the acquisition of skills, including cognitive skills and non-cognitive skills, that are crucial to one’s lifetime achievements [23,26,27,28,29]. For another, since at this stage, children are too young (under two years old) to enter school and society, there are few unobserved changes in the environment over time, so this enables the focus to be placed on the family itself, without having to consider the impact of other external factors. Therefore, investigating the sibling effects on children’s early development contributes to the establishment of a purely causal relationship with the Q–Q trade-off.

Additionally, there is another advantage to using a sample that comprises infants below the age of 2: it facilitates the identification of the mechanism driving sibling effects; that is, it can be more easily determined whether having siblings exerts an impact on the intrahousehold resource allocation and, in turn, affects the newborn’s early development. Parents’ investment has been previously emphasized [1], but it is poorly identified in much of the literature [24]. The home environment and parental warmth are two crucial resources that provide a sound foundation for early childhood development [30,31,32,33]. Evidence has been found that an additional child substantially reduced parental time [24].

Differing from other work studying the trade-off, the uniqueness of this paper is represented by the following four aspects:

First, in terms of the target, we investigated sibling effects on infants’ early development in rural families in western China. Compared with families from coastal cities, inland rural ones are more vulnerable to resource constraints in China [34,35,36]. Investigating the trade-off and intrahousehold resource allocation underlying it enables us to get a deeper insight into the urban–rural inequality in the human capital of China, and understanding this issue is vital for China’s future growth [37,38].

Second, the samples in this study were infants younger than 2 years old. Contrary to studies that focused on the impact of additional children and held the opinion that the birth order was important [6,8], what we studied here was the sibling effects on the “newcomer” baby. Since a newborn baby younger than two years old is too young to have another younger sibling, the birth order does not matter in this study.

Third, in terms of the approach, considering that sometimes the external validity of the IV is difficult to satisfy, we took another approach called the matching-smoothing (MS) method to solve selection bias [39]. The procedure of matching-smoothing (MS) is described in Section 3.3 in more detail. As its name indicates, MS is composed of two parts: matching and smoothing. The matching part is just propensity score matching (PSM) in which the treatment unit (have siblings) is one-to-one matched to the comparison unit (no siblings) on the basis of their propensity scores. In the smoothing part, the trend of matched differences or unobserved selection is fitted by a non-parametric smoothing model using local polynomial regression.

Last, as for the mechanism, our paper also differs from the study of Juhn et al. that only focused on parental time [24]. The links of the home environment and parental warmth to children’s early development have been found to be robust and positive [30,33,40,41,42,43]. In this paper, home environment means the overall quality of the child care environment as measured by the total score of the Child Care Home Observation for Measurement of the Environment (CC-HOME) inventory. The CC-HOME inventory contains 43 caregiver-report binary-choice items in six subscales [44]. Parental warmth means the parents’ warm affectionate behaviors toward their baby as measured by the primary caregiver’s responses to six relevant questions [45]. In comparison, the home environment provides general information about the child care environment, whereas parental warmth directly assesses the parents’ warm affectionate behaviors in detail. They are described in Section 3.4 in more detail, and the items or questions used to measure them are presented in Table A1 and Table A2. With the findings that having siblings causes parents to spread out their investment in both the home environment and parental warmth for children, our work sheds light on the relation between the Q–Q trade-off and early childhood development.

The rest of this paper is organized as follows. Section 2 introduces our hypothesis and explains the identification strategies we applied to verify it. Section 3 describes the data and variables. Section 4 reports the empirical results. Section 5 discusses our main results. Section 6 concludes the paper.

## 2. Hypotheses

The purpose of our study is to investigate the direct association between having siblings and a newborn infant’s early development, which was measured by their cognitive, language, motor, and social-emotional skills. We put forward the first hypothesis as follows:

**Hypothesis** **1.** *Having siblings has impacts on the infant’s early development.*

In terms of the mechanism, the home environment and parental warmth are two important issues in the literature on early childhood development [33]. More specifically, the home environment builds a foundation for children’s skill formation [30,40,41], and parental warmth is crucial for children’s early outcomes [42,43]. As a matter of fact, parents’ investment has been poorly measured and estimated in many studies, yet it also lies at the center of the Q–Q theory. Hence, our second hypothesis is as follows:

**Hypothesis** **2a.** *Having siblings influences the parents’ investment in the home environment.*

**Hypothesis** **2b.** *Having siblings influences the parents’ investment in parental warmth.*

## 3. Empirical Strategy

### 3.1. Sampling

The data we used in this paper were collected from a sample of rural families in Guizhou province, a typical underdeveloped and minority-inhabited area in western China. In Guizhou, one developing prefecture was randomly chosen as the sample prefecture from which the program randomly selected one county. Similarly, after that, one town was randomly chosen from the list of all towns in the county. The sample town consists of nine villages in total.

### 3.2. Survey Organization

The survey was conducted in Guizhou province in 2017 by the China Reach program of the China Development Research Foundation (CDRF). We first obtained a list of registered births in each village of the sample town from the local regulatory authority. From the list, we identified all households that had babies who were under 24 months old at the time of the survey. We ended up with 446 households that met the criteria. After the sample households were selected, we visited them to conduct one-on-one interviews. After dropping 2 sample households with missing information, our final sample includes 444 infants from nine villages.

The Peking University Institutional Review Board (PU IRB), Beijing, China, approved the ethical assessment of the study (No. IRB00001052-17056), and verbal informed consent was obtained from all study subjects.

### 3.3. Econometric Model

We used the multivariate regression analysis to test Hypothesis 1 as follows:

(1) $$bayley_{i}=\beta_{0}+\beta_{1}sibling_{i}+\beta_{2}X_{1i}+\tau_{i}+\varepsilon_{1i}$$

where *bayley_i_* denotes the development of infant *i* measured by the Bayley Scales of Infant and Toddler Development III (Bayley-III) and comprises four variables: *cog_i_*, *lang_i_*, *motor_i_*, and *soemo_i_*, which are described in detail in the next section. *sibling_i_* is a dummy variable to represent whether the infant has siblings (*sibling_i_* = 1) or not (*sibling_i_* = 0). *X*_1*i*_ denotes infant and family characteristics, including gender (*male*), age in months (*month*), infants’ birthweight (*birthweight*), infants’ birth-height (*birthheight*), parents’ age (*fage*, *mage*), employment status (*fwork*, *mwork*), education (*fedu*, *medu*), minority (*fminority*, *mminority*), whether the mother is the primary caregiver (*moncare*), and the family income groups. $\tau_{i}$ includes the village dummy variable to control for the unobserved heterogeneity at the village level. $\varepsilon_{1i}$ is the error term, and it is assumed to be *i.i.d*. Since, in the relevant literature, parental socioeconomic status [46], family income [40,47,48], and community environment have been found to be associated with early childhood development [49,50], we controlled for these covariates in the baseline regression.

However, as stated above, selection bias is the main challenge. It can be described by the following equation:

(2) $$E\left(bayley_{1i}|sibling_{i}=1\right)-E\left(bayley_{0i}|sibling_{i}=0\right)\;=E\left(bayley_{1i}|sibling_{i}=1\right)-E\left(bayley_{0i}|sibling_{i}=1\right)\;+E\left(bayley_{0i}|sibling_{i}=1\right)-E\left(bayley_{0i}|sibling_{i}=0\right).$$

The OLS estimate is the left-side item of the equation. The first item on the right-hand side is the average treatment effect on the treated (ATT), and this is what we are interested in. However, the second item on the right-hand side, the selection bias, potentially contaminates the estimate.

In order to solve this problem, or at least partly, we applied the MS method to evaluate the heterogeneous treatment effect of siblings. The matching part of this algorithm is the same as in PSM [51]. According to the dummy *sibling* variable, the sample was divided into the treatment group (have siblings) and the control group (no siblings). Then, the probit regression model was used to estimate the propensity score for all families, namely, their probability of having more than one child, given all the observed covariates. After that, on the basis of the estimated propensity score, using the nearest-neighbor matching method, the treatment unit was one-to-one matched with the control unit; as a consequence, in each pair, a treated unit was matched with a control unit having almost the same propensity sco. However, PSM is often sensitive to the unobserved selection.

To address the problem, in the smoothing part, the matched differences between the treated and controlled units were plotted, and the local polynomial regression—a non-parametric smoothing model—was used to fit the trend of matched differences against the propensity score [52]. That is, the MS approach yields the treatment effect heterogeneity or unobserved selection as a non-parametric representation rather than the imposed functional form.

To test Hypothesis 2, we used the multivariate regression as follows:

(3) $$home_{i}\left(warmth_{i}\right)=\gamma_{0}+\gamma_{1}sibling_{i}+\gamma_{2}X_{2i}+\tau_{i}+\varepsilon_{2i}$$

where *home_i_* measures the home environment of infant *i*, and *warmth_i_* measures the parental warmth toward infant *i*. They are also described in detail in the next section. *X*_2*i*_ controls for family characteristics as in Equation (1), $\tau_{i}$ still controls for village heterogeneity, and $\varepsilon_{2i}$ is the error term and is assumed to be *i.i.d*. Likewise, to get rid of the influence of selection bias, we adopted the MS method again to estimate Equation (3) using all the observed covariates of the family and village characteristics mentioned above.

### 3.4. Measurement of Key Variables

The dependent variable in the baseline regression, *bayley*, is the development of children measured by the Bayley Scales of Infant and Toddler Development III (Bayley-III), a well-known scale that has great reliability and validity. This standardized measurement, originally developed by Nancy Bayley [53], contains a series of play tasks and questions, and the scale scores are internationally applied to evaluate the developmental functioning of infants/toddlers from birth to age 3 [54].

In this study, we adopted its four main subtests—the cognitive, language, motor, and social-emotional scales—to assess the children’s cognitive skills (such as playing, attention to objects, and counting), language skills (such as understanding and expression of language), motor skills (such as fine and gross motor skills), and social-emotional skills (such as social responsiveness and self-regulation), respectively. They are represented by the variables, *cog_i_*, *lang_i_*, *motor_i_*, and *soemo_i_*. All enumerators attended a week-long training course on how to administer the Bayley-III, and they were blind to the study hypotheses. They administered the test one-on-one with household members using a standardized set of toys and a detailed scoring sheet. The assessments of cognitive, language, and motor skills depend on scores that are given according to the infant’s successful completion of the items, while the social-emotional score comes from caregivers’ responses to relevant questions. A lower score usually means a higher risk of children experiencing developmental problems in the future [55].

In order to assess the home environment and parental warmth, trained enumerators made a 90–120-minute home visit when the infant and the primary caregiver were both present and the infant was awake. The home environment was assessed by the infant/toddler version of the CC-HOME inventory designed by Bradley et al. [44]. It includes 43 caregiver-report binary-choice items in six subscales: Caregiver Responsivity, Acceptance, Organization, Learning Materials, Caregiver Involvement, and Variety of Stimulation. The items and internal consistencies in terms of Cronbach’s alpha are presented in detail in Table A1. The Cronbach’s alpha coefficients of the HOME inventory and its six subscales are all larger than 0.7, implying that the internal consistencies are acceptable with this study’s sample. The total score for the home environment, i.e., the home variable used in our mechanism analysis, is the sum of the scores for the six subscales.

Parental warmth was assessed by using the primary caregiver’s responses to the six questions on how often they showed warm affectionate behaviors to their babies. The six questions take less time and training to administer than most existing measures and are strongly robust and reliable [45]. The questions and internal consistencies are presented in Table A2. The Cronbach’s alpha of parental warmth is equal to 0.8, implying its internal consistency is good in this sample, too. The response to each question is on a five-point scale, where 1 indicates never/almost never and 5 indicates always/almost always. The total score for parental warmth, i.e., the warmth variable used in our mechanism analysis, is the sum of scores for the six questions. The lowest possible score is 6 (lowest warmth) and the full score is 30 (highest warmth).

## 4. Empirical Results

### 4.1. Descriptive Statistics

**Figure A1:**
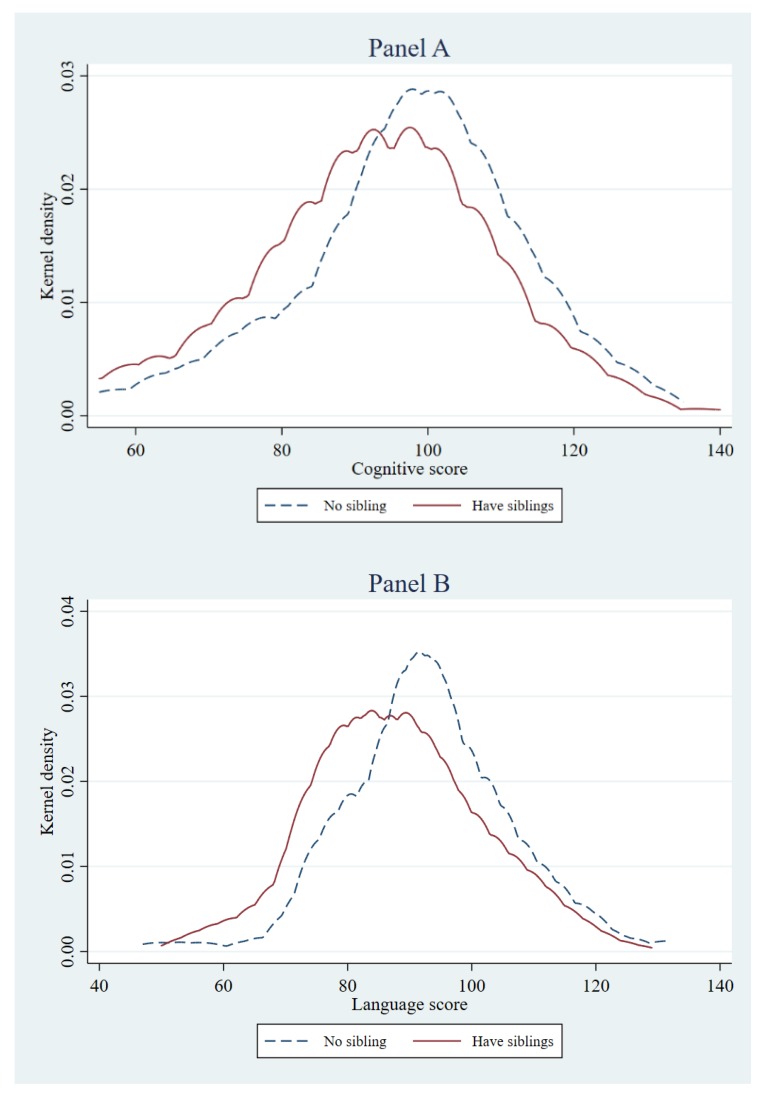

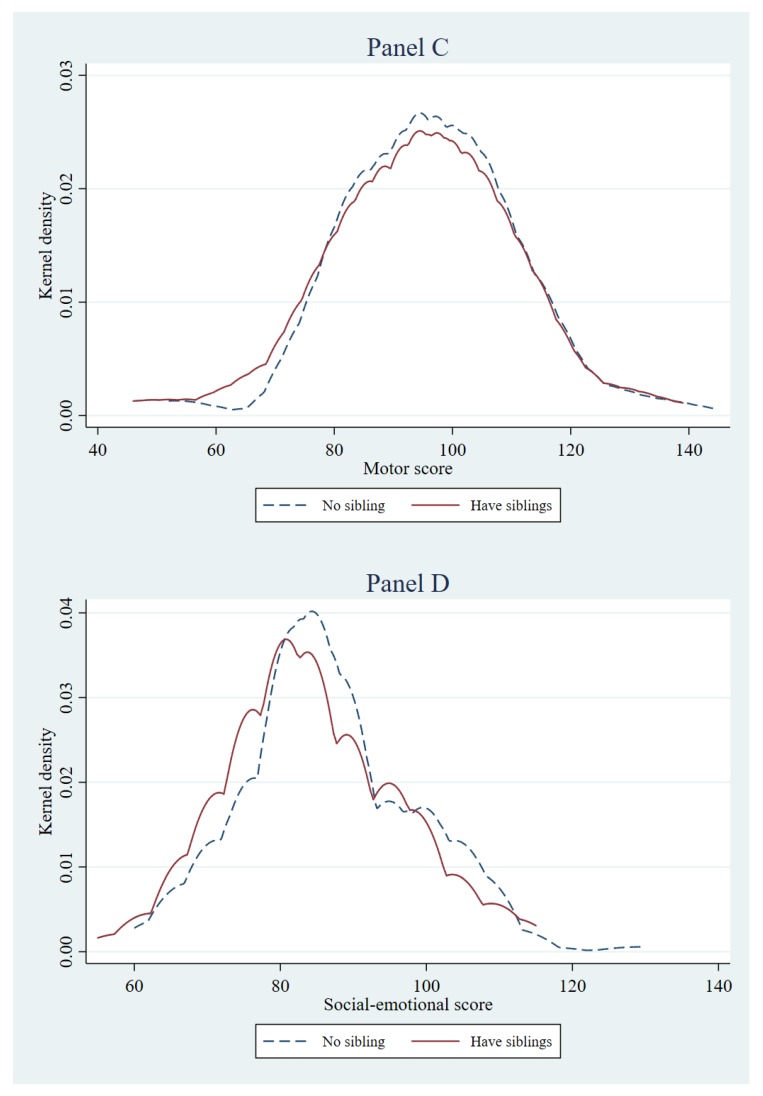
The density distribution of cognitive score (Panel A), language score (Panel B), motor score (Panel C), and social-emotional score (Panel D) of the treatment group (have siblings, solid line) and the control group (no sibling, dashed line).

The distribution of the Bayley-III score (Figure A1) leaves us with an intuitive impression of the developmental differences between the treatment group (have siblings) and the control group (no siblings). The mean of the cognitive, language, and social-emotional score in the one-child family is higher, while the difference in the means of the motor scores is not obvious. It implies a negative association between having siblings and an infant’s development of cognitive, language, and social-emotional skills.

Descriptive statistics report the sample mean and standard deviation of each variable (Table A3). The Wald test shows that the differences in the average cognitive, language, and social-emotional scores are statistically significant. Hence, we have reason to believe that an infant’s neurodevelopment (except the motor skill) indeed has a negative correlation with having siblings. The Q–Q trade-off makes sense in this context. Further, the means of the control variables—age and year of schooling of parents, father’s employment status, and family’s total income in the last 12 months between 100,000 and 250,000 yuan—all show a significant gap between the two groups. Finally, in the mechanism analysis, the scores for the dependent variables—*home* and *warmth*—are also higher in the no-sibling group on average, indicating that the home environment and parental warmth are both negatively associated with having siblings, too.

### 4.2. The Sibling Effects on the Infant’s Development

Table 1 presents the estimates for the sibling effects on the infant’s Bayley score. In Panel A, after controlling for the infant’s characteristics (Column 2), family’s characteristics (Column 3), and village fixed effects (Column 4), successively, the OLS-estimated negative impact of having siblings on the cognitive score is still statistically significant. Therefore, the baseline regressions show that, after controlling for other factors, the infant’s cognitive score in the have-sibling group is lower than that in the no-sibling group on average.

Then, in order to overcome selection bias, we applied the PSM method to estimate the sibling effects. Column 5 presents the PSM estimate of the effect of having siblings on the cognitive score for the ATT analysis, and it can be compared with the MS estimate shown in Column 6. Both the PSM and MS estimates indicate that the negative effect of having siblings on an infant’s cognitive score is highly significant. Furthermore, owing to selection bias, the OLS severely underestimates the absolute value of sibling effects. The MS-estimated coefficient of the sibling effect on the cognitive score is −0.19, which is closer to a small effect size than it is to the trivial one implied by the OLS estimation.

The estimations of the sibling effects on infants’ language, motor, and social-emotional scores are presented in Panels B–D in Table 1, respectively. Similarly, the estimated sibling effects on infants’ language and social-emotional score are also significantly negative, and selection bias drives the OLS to underestimate the sibling effects according to the PSM and MS estimations. However, little evidence is found that supports the assertion that siblings have an adverse impact on an infant’s motor skills. In terms of the effect size of having siblings, it is small on the infant’s language score (−0.21) and trivial on the infant’s social-emotional score (−0.14).

**Figure A2:**
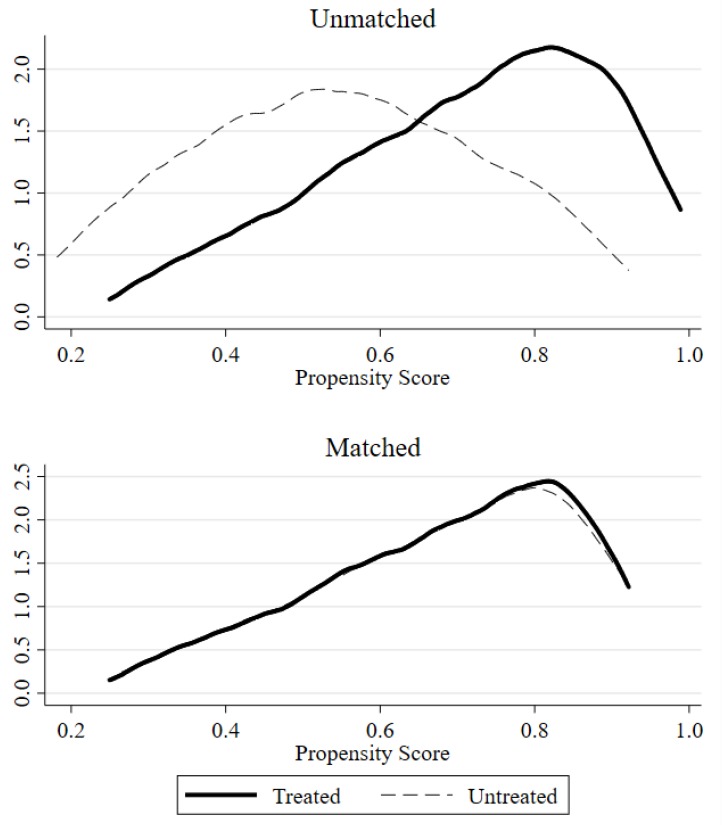
The density distribution of propensity score before and after matching.

**Figure A3:**
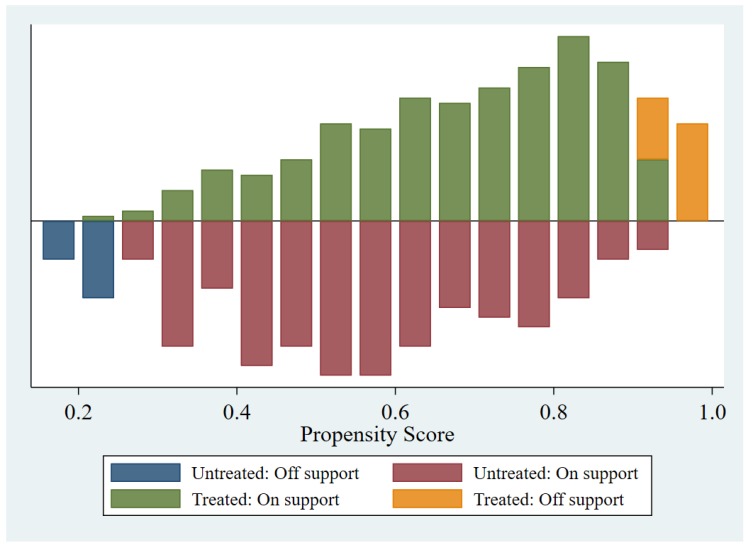
The common support of the propensity score.

Figure A2 and Figure A3 show the density distribution and the common support of the propensity score before and after matching in the PSM analysis. Before matching, there is a huge difference in the distribution between the treatment group and the control group. After matching, the difference diminishes substantially, and the two distribution curves almost coincide. There is a large common support between the two groups according to Figure A3. Most observations favor support, which means that the common support assumption of the PSM method is satisfied, or in other words, the matching result is satisfactory.

**Figure 1:**
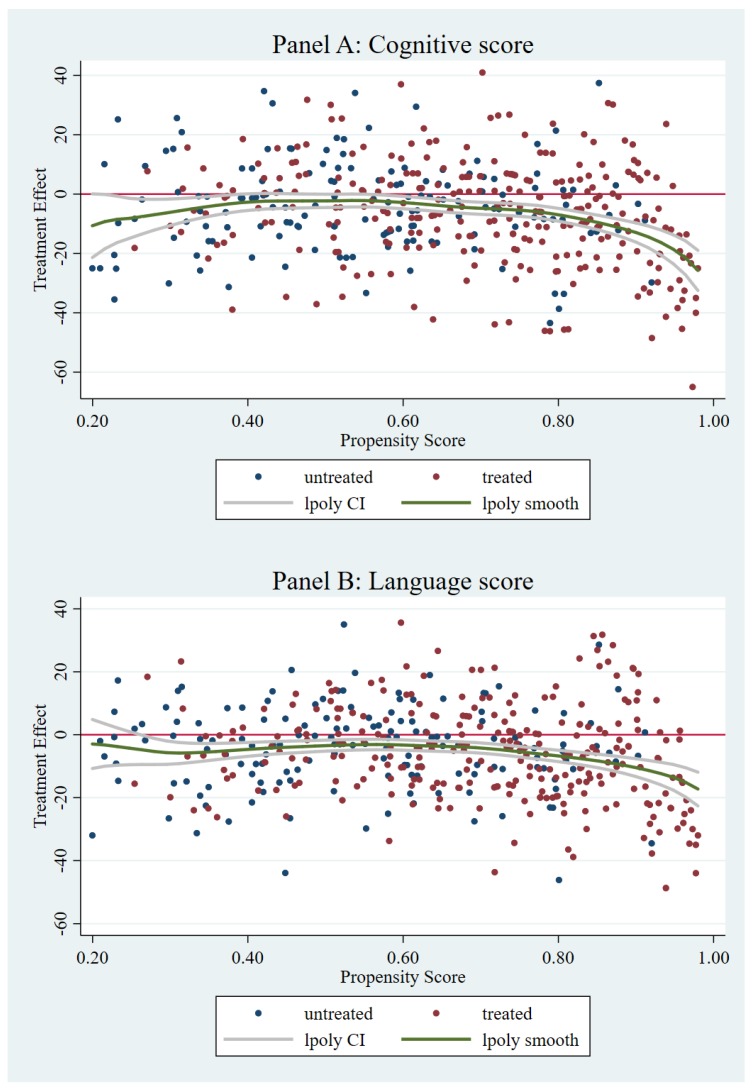

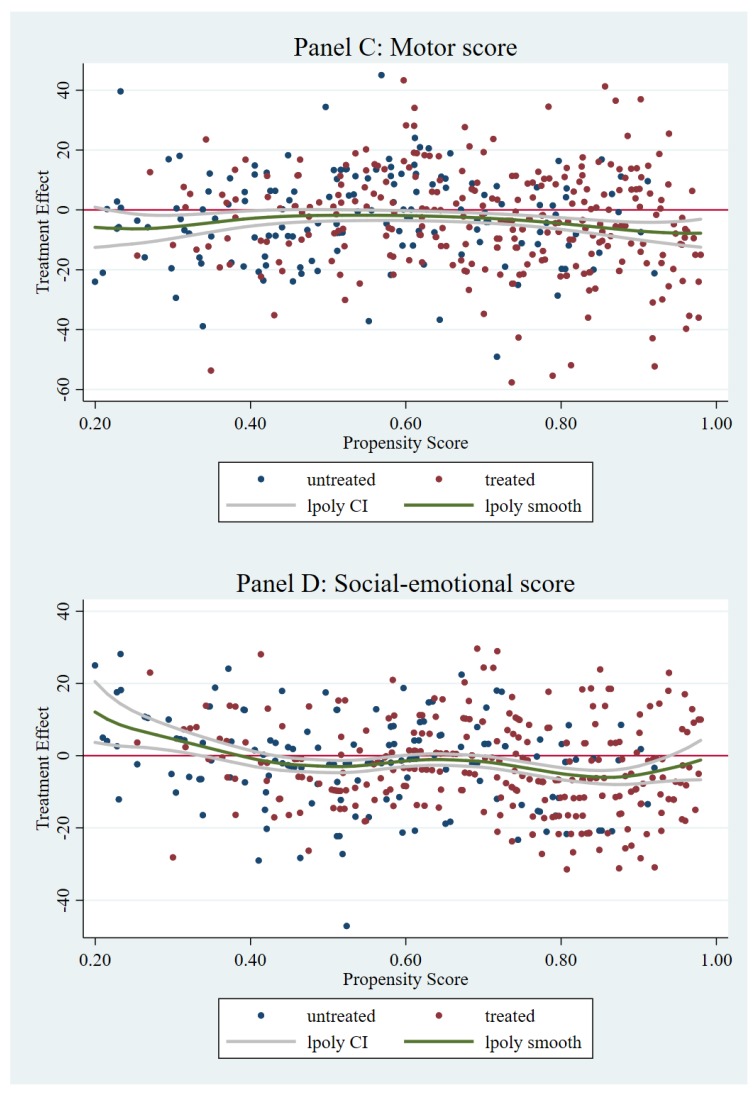
The heterogeneous effect of siblings on infants’ cognitive, language, motor, and social-emotional score (matching-smoothing (MS) method).

Figure 1 demonstrates the advantage of the MS method by using the local polynomial regression to fit the matched differences. The figure plots the heterogeneous treatment effect in a non-parametric representation. It overwhelms the prior imposed functional form of PSM because of the unobserved selection. In Figure 1, the X-axis represents the continuous propensity score, and the Y-axis describes differences in the infants’ expected development scores. We can observe that the traditional assumption of a linear function in PSM is not reasonable here. There are progressively negative sibling effects on infants’ cognitive, language, and social-emotional scores as the propensity for having siblings increases (Figure 1, Panels A, C, and D).

Figure 1 implies that the selection bias is considerably serious for families with a greater propensity to have more than one child according to the observed characteristics. For those families, the selection plays a more pivotal role in affecting both their fertility decision and infant’s development.

In short, in terms of early childhood development, having siblings exerts a negative influence on an infant’s neurodevelopment, including cognitive, language, and social-emotional skills, in rural families in Guizhou, China, and these effects might be detrimental to these children’s future development in many aspects. Overall, the Q–Q trade-off holds from the perspective of those newborn babies, and Hypothesis 1 is verified.

### 4.3. The Mechanism behind the Trade-Off

In this section, the mechanisms through which having siblings affects the infant’s neurodevelopment are discussed. The discussion is focused on the sibling effects on the home environment and parental warmth, as the influences of these two factors on early childhood development have been emphasized in many studies [31]. We included the infant and family characteristics and village dummy variables as the covariates of the home environment and parental warmth.

Table 2 reports the estimates for the sibling effects on the home environment and parental warmth. First, the OLS, PSM, and MS estimates are all significantly negative, which is reasonable. Faced with an increase in the number of children, parents in rural areas, who are constrained by a limited budget, have to reallocate intrahousehold resources. As a result, their investments in the home environment and warm affectionate behaviors decline. Second, the selection bias is more serious in the estimate for sibling effects on the home environment, which is also clearly depicted in Table 2. In short, two key mechanisms are successfully identified. Having siblings hinders the infant’s neurodevelopment by lowering the parents’ investment in the home environment and parental warmth.

### 4.4. Mediation Analysis

The causal mediation analysis is a three-stage linear regression as follows:

(4) $$bayley_{i}=\alpha_{0}+\alpha_{1}sibling_{i}+\varepsilon_{1i}$$

(5) $$home_{i}\left(warmth_{i}\right)=\gamma_{0}+\gamma_{1}sibling_{i}+\varepsilon_{2i}$$

(6) $$bayley_{i}=\theta_{0}+\theta_{1}sibling_{i}+\theta_{2}home_{i}\left(warmth_{i}\right)+\varepsilon_{3i}$$

The definitions of the variables are the same as in Equations (1) and (3). To avoid unnecessary multicollinearity, here we only identify the pure mediation effect. That is, we did not include any infant and family characteristics or village dummy variables in the mediation effect model, as in the other study [56].

Table 3 presents the estimates for the mediation effect of the home environment. The first stage is the baseline model, so the resulting estimate is the same as the result from the univariate analysis. The sibling effects on infants’ developmental skills (except the motor score) are all negative and significant (Row 1, Table 3). The second stage is the mediation model, from which we can see that, as observed in Table 2, having siblings has a substantially adverse impact on the home environment (Row 2, Table 3). In the third stage, the comprehensive model, the sibling and home environment coefficients are both statistically significant in determining cognitive, language, and social-emotional scores (Row 3, 4, Table 3), and the sibling coefficient is lower than that in the baseline model.

Furthermore, as the Sobel test has noted limits and flaws [57], we used the bias-corrected bootstrap test [58,59] with 95% confidence intervals to examine its mediation effect. Results from meditational analyses show that the home environment indeed acts as a mediator of the sibling effects on infants’ cognitive and language scores, particularly on the language skill. This can also be inferred in the mediated proportion of total effect (Rows 7–9, Columns 1–2, Table 3). Likewise, the estimates of the mediation effect of parental warmth confirm our hypotheses (Table 4). This channel plays a statistically significant role in sibling effects on infants’ language and social-emotional skills (Rows 7, 8, Column 2, 4, Table 4), particularly on the latter, as the mediated proportion of its total effect is 17.3% (Row 9, Column 4, Table 4).

### 4.5. Sibling Number Effects

The key independent variable in the multivariate analysis above, *sibling*, is a dummy variable, so it only has one of two outcomes, namely, whether the infants have siblings or not. The sibling number effect is also an interesting issue related to the Q–Q trade-off. However, because of the One-Child Policy, although relaxed recently, most families in China usually have two or three children at most, i.e., the sibling number is one or two in most of the sample population.

Here, we replaced the variable *sibling* with *num_sibling* and did the multivariate analysis again. Table A8 presents the estimates for the sibling number effects on infants’ developmental skills. The results are consistent with the estimates for the sibling effects in Table 1. The increase in the sibling number also considerably decreases the infant’s cognitive, language, and social-emotional score.

Then, we used the variable *num_sibling* to estimate the mediation effect model again to check the robustness of the mediation effect. Table A9 and Table A10 report the estimates for the mediation effect of the home environment and parental warmth, respectively. The home environment works as a key mediator of sibling number effects on the infant’s cognitive and language skills, and parental warmth plays a vital mediator role in sibling number effects on the infant’s social-emotional skills. It is close to the results reported in Table 3 and Table 4. The mechanism identification is robust, too.

## 5. Discussion

In this paper, we discuss the relation between the famous Q–Q trade-off theory and early childhood development in rural Chinese families. Using survey data collected from families in Guizhou province in 2017, we applied a multivariate analysis to investigate the sibling effects on infants’ cognitive, language, motor, and social-emotional skills, which were measured by the well-known Bayley-III scale score.

First, the OLS estimates reveal that, after controlling for the infant’s characteristics, family characteristics, and village fixed effects, sibling effects on infants’ neurodevelopment (except motor skill) are all negative and statistically significant. Second, the PSM and MS estimates show that having siblings indeed exerts adverse impacts on infants’ cognitive, language, and social-emotional skills, but the OLS estimate tends to underestimate this effect because of selection bias. In terms of the magnitude, the real sibling effect sizes on infants’ cognitive and language skills are small, and the effect sizes on social-emotional skills are trivial. Third, two key mechanisms are successfully identified here: the home environment and parental warmth. The home environment plays an essential role in sibling effects on infants’ cognitive and language scores, while parental warmth plays a vital part in sibling effects on infants’ language and social-emotional score. Finally, the sibling number effect on infants’ neurodevelopment is consistent with the sibling effects and thus verifies the robustness of the estimates.

Given the multi-factorial measure of the home environment, we further examined which subscale is most strongly correlated with infants’ neurodevelopment. When looking at an infant’s cognitive skills, the results from our analyses show that acceptance, organization, learning materials, and variety of stimulation have significantly positive effects (Table A11, Table A12 and Table A13). When compared, learning materials have the largest effect size. When looking at an infant’s language skills, our results show that five out of the six scales have significant effects. The only exception is acceptance. In terms of the magnitude of the estimated coefficient, organization is the biggest, followed by learning materials, and the smallest is involvement. When looking at an infant’s social-emotional skills, we find that only the learning materials come out as statistically significant. Taken together, our study provides new evidence that learning materials might be the most effective home environment factor for improving the comprehensive neurodevelopment of infants.

Compared with their urban counterparts, Chinese rural households are more vulnerable to resource constraints. Thus, having an additional child will tend to decrease parents’ investment in the home environment and parental warmth for the individual child on average. Qin et al. (2018) found evidence of the Q–Q trade-off in lower-income and less-developed credit market areas using the 2005 inter-census 1% population survey data in China, and they only observed evidence in lower-income and less-developed credit market areas [60]. Considering the fact that the lower-income and less-developed credit market is mainly a rural phenomenon, our finding is consistent with that of Qin et al. (2018).

We acknowledge a couple of limitations of our study. First and foremost, our sample is not nationally representative, so the conclusions may not be generally applicable to all families in the whole country. As a consequence of the lack of an urban sample, we are unable to examine whether the Q–Q trade-off still holds for urban families with higher income and a more developed credit market. Second, the impact of household resource constraints on the relationship between the Q–Q trade-off and early childhood development could be worth investigating. We leave this issue for future research.

## 6. Conclusions

Despite the limitations, all of these findings support the conclusion that the Q–Q trade-off holds in rural families in China’s Guizhou province. Having siblings indeed hinders a newborn’s early development in this setting, and it is harmful to their future achievements. The important mechanism driving the trade-off is the reallocation of intrahousehold resources.

We believe that this conclusion is of certain value to local and even national policymakers who are facing the knotty challenge of substantial urban–rural inequality in human capital in today’s China.

## Figures and Tables

**Table 1:** The estimates for sibling effects on infants’ Bayley score.

Bayley Score	OLS	OLS	OLS	OLS	PSM	MS
	(1)	(2)	(3)	(4)	(5)	(6)
Panel A: Cognition score						
*sibling*	−4.88 *** (1.60)	−4.43 *** (1.28)	−3.22 ** (1.42)	−3.91 ** (1.67)	−5.81 *** (2.11)	−6.46 *** (2.15)
standardized coefficient	−0.14	−0.13	−0.09	−0.11	−0.17	−0.19
two-tailed *p*-value	0.002	0.001	0.03	0.03	0.01	0.006
infant’s characteristics		Yes	Yes	Yes		
family’s characteristics			Yes	Yes		
village fixed effect				Yes		
Panel B: Language score						
*sibling*	−5.15 *** (1.35)	−5.06 *** (1.08)	−3.99 *** (1.09)	−4.22 *** (1.32)	−4.97 ** (2.01)	−5.94 *** (1.83)
standardized coefficient	−0.18	−0.18	−0.14	−0.15	−0.17	−0.21
two-tailed *p*-value	0.000	0.000	0.001	0.003	0.02	0.003
infant’s characteristics		Yes	Yes	Yes		
family’s characteristics			Yes	Yes		
village fixed effect				Yes		
Panel C: Motor score						
*sibling*	−1.83 (1.57)	−2.35 * (1.25)	−1.32 (1.37)	−2.17 (1.44)	−3.14 (2.27)	−3.58 (2.09)
standardized coefficient	−0.06	−0.07	−0.04	−0.07	−0.09	−0.11
two-tailed *p*-value	0.13	0.07	0.34	0.14	0.18	0.11
infant’s characteristics		Yes	Yes	Yes		
family’s characteristics			Yes	Yes		
village fixed effect				Yes		
Panel D: Social-emotional score						
*sibling*	−2.51 ** (1.17)	−2.63 *** (0.97)	−2.19 ** (0.91)	−2.14 ** (0.85)	−4.03 ** (1.75)	−3.48 ** (1.41)
standardized coefficient	−0.10	−0.11	−0.09	−0.09	−0.16	−0.14
two-tailed *p*-value	0.03	0.01	0.02	0.02	0.03	0.02
infant’s characteristics		Yes	Yes	Yes		
family’s characteristics			Yes	Yes		
village fixed effect				Yes		
Observation	444	444	444	444	432	432

Note: (i) Coefficients and standard errors are reported to the nearest 0.01. (ii) In the ordinary least squares (OLS) estimate, the robust standard errors clustered at the village level are presented in parentheses. In the propensity score matching (PSM) and matching-smoothing (MS) estimates, average treatment effect on the treated (ATT) is reported, and the robust standard errors are obtained by the bootstrapped method with 50 replications. (iii) *, **, and *** denote *p* < 0.1, *p* < 0.05, *p* < 0.01 in two-tailed tests, respectively. (iv) The estimates for covariates are presented in detail in Table A4, Table A5, Table A6 and Table A7.

**Table 2:** The estimates for sibling effects on the home environment and parental warmth.

Intermediate Variable	*Home*	*Home*	*Home*	*Warmth*	*Warmth*	*Warmth*
	OLS	PSM	MS	OLS	PSM	MS
	(1)	(2)	(3)	(4)	(5)	(6)
*sibling*	−0.62 ** (0.28)	−1.21 * (0.70)	−1.28 ** (0.60)	−0.82 ** (0.38)	−1.01 ** (0.47)	−0.84 ** (0.41)
two-tailed *p*-value	0.04	0.10	0.04	0.04	0.04	0.05
infant characteristics	Yes	Yes	Yes	Yes	Yes	Yes
family characteristics	Yes	Yes	Yes	Yes	Yes	Yes
village fixed effect	Yes	Yes	Yes	Yes	Yes	Yes
Observation	444	432	432	444	432	432

Note: (i) Coefficients and standard errors are reported to the nearest 0.01. (ii) In the OLS estimate, the robust standard errors clustered at the village level are presented in parentheses. In the PSM and MS estimates, ATT is reported, and the robust standard errors are obtained by the bootstrapped method with 50 replications. (iii) *, ** denote *p* < 0.1, *p* < 0.05 in two-tailed tests, respectively. (iv) The village fixed effect is controlled by 8 village dummies.

**Table 3:** The estimates for the mediation effect of the home environment.

**Bayley Score**	***Cog***	***Lang***	***Motor***	***Soemo***
*sibling*	−4.88 *** (1.60)	−5.15 *** (1.35)	−1.83 (1.57)	−2.51 ** (1.17)
**Intermediate variable**	***home***	***home***	***home***	***home***
*sibling*	−1.34 ** (0.53)	−1.34 ** (0.53)	−1.34 ** (0.53)	−1.34 ** (0.53)
**Bayley score**	***cog***	***lang***	***motor***	***soemo***
*sibling*	−4.46 *** (1.61)	−4.47 *** (1.33)	−1.31 (1.57)	−2.26 * (1.18)
*home*	0.32 ** (0.14)	0.51 *** (0.12)	0.39 *** (0.14)	0.19 * (0.11)
Sobel test	−0.42 * (0.25)	−0.68 ** (0.31)	\	−0.25 (0.17)
bootstrap test	−0.42 * (0.24)	−0.68 ** (0.30)	\	−0.25 (0.19)
percentile confidence interval of indirect effect	(−0.98, −0.04)	(−1.36, −0.13)	\	(−0.65, 0.05)
bias-corrected confidence interval of indirect effect	(−1.09, −0.09)	(−1.46, −0.20)	\	(−0.72, 0.02)
mediated proportion of total effect	8.7%	13.2%	\	10.1%

Note: (i) Coefficients and standard errors are reported to the nearest 0.01. (ii) The robust standard errors clustered at the village level are presented in parentheses. (iii) *, **, and *** denote *p* < 0.1, *p* < 0.05, *p* < 0.01 in two-tailed tests, respectively. (iv) The bootstrap test is based on resampling with 1000 replications; a 95% confidence interval is reported. (v) *Cog*: Cognitive score; *Lang*: Language score; *Motor*: Motor score; *Soemo*: Social-emotional score.

**Table 4:** The estimates for the mediation effect of parental warmth.

**Bayley Score**	***Cog***	***Lang***	***Motor***	***Soemo***
*sibling*	−4.88 *** (1.60)	−5.15 *** (1.35)	−1.83 (1.57)	−2.51 ** (1.17)
**Intermediate variable**	***warmth***	***warmth***	***warmth***	***warmth***
*sibling*	−0.99 *** (0.36)	−0.99 *** (0.36)	−0.99 *** (0.36)	−0.99 *** (0.36)
**Bayley score**	***cog***	***lang***	***motor***	***soemo***
*sibling*	−4.75 *** (1.62)	−4.80 *** (1.35)	−1.80 (1.57)	−2.08 * (1.18)
*warmth*	0.13 (0.21)	0.36 ** (0.18)	0.02 (0.21)	0.44 *** (0.15)
Sobel test	−0.13 (0.22)	−0.35 * (0.22)	\	−0.43 ** (0.22)
bootstrap test	−0.13 (0.22)	−0.35 * (0.21)	\	−0.43 ** (0.23)
percentile confidence interval of indirect effect	(−0.65, 0.28)	(−0.84, −0.03)	\	(−0.94, −0.06)
bias-corrected confidence interval of indirect effect	(−0.71, 0.23)	(−0.96, −0.06)	\	(−1.05, −0.10)
mediated proportion of total effect	2.7%	6.8%	\	17.3%

Note: (i) Coefficients and standard errors are reported to the nearest 0.01. (ii) The robust standard errors clustered at the village level are presented in parentheses. (iii) *, **, and *** denote *p* < 0.1, *p* < 0.05, *p* < 0.01 in two-tailed tests, respectively. (iv) The bootstrap test is based on resampling with 1000 replications; a 95% confidence interval is reported. (v) *Cog*: Cognitive score; *Lang*: Language score; *Motor*: Motor score; *Soemo*: Social-emotional score.

## Appendix A

**Table A1:** The infant/toddler version of the Child Care Home Observation for Measurement of the Environment (CC-HOME) inventory.

Subscale	Cronbach’s Alpha
**Responsivity**	**0.82**
1. Caregiver spontaneously vocalizes to child at least twice.	0.80
2. Caregiver responds verbally to child’s vocalizations or verbalizations.	0.80
3. Caregiver tells child the name of object or person during visit.	0.81
4. Caregiver’s speech is distinct, clear, and audible.	0.80
5. Caregiver initiates verbal interchanges with visitor.	0.79
6. Caregiver converses freely and easily.	0.81
7. Caregiver permits child to engage in messy play.	0.79
8. Caregiver spontaneously praises child at least twice.	0.80
9. Caregiver’s voice conveys positive feelings toward child.	0.83
10. Caregiver caresses or kisses child at least once.	0.80
11. Caregiver responds positively to praise of child offered by visitor.	0.81
**Acceptance**	**0.79**
12. Caregiver does not shout at child.	0.79
13. Caregiver does not express overt annoyance with or hostility to child.	0.71
14. Caregiver neither slaps or spanks child during visit.	0.76
15. No more than one instance of physical punishment during past week.	0.72
16. Caregiver does not scold or criticize child during visit.	0.65
17. Caregiver does not interfere with or restrict child three times during visit.	0.77
18. At least 10 books are present and visible.	0.84
**Organization**	**0.71**
19. Caregiver is one of no more than three regular substitutes used for child.	0.69
20. Child is taken on an outing at least once a week.	0.71
21. Child gets out of house at least four times a week.	0.64
22. Caregiver has an emergency medical and/or accident plan.	0.66
23. Child has a special place for toys and treasures.	0.65
24. Child’s play environment is safe.	0.76
**Learning Materials**	**0.86**
25. Muscle activity toys or equipment.	0.79
26. Push or pull toy.	0.85
27. Stroller or walker, kiddie car, scooter, or tricycle.	0.82
28. Caregiver provides toys for child to play with during the visit.	0.78
29. Cuddly toy or role-playing toys.	0.80
30. Learning facilitators—mobile, table and chair, high chair, playpen.	0.82
31. Simple eye–hand coordination toys.	0.91
32. Complex eye–hand coordination toys.	0.82
33. Toys for literature and music.	0.87
**Involvement**	**0.76**
34. Caregiver keeps child in visual range, looks at often.	0.71
35. Caregiver talks to child while doing household work.	0.69
36. Caregiver consciously encourages developmental advance.	0.64
37. Caregiver invests maturing toys with value via personal attention.	0.65
38. Caregiver structures child’s play periods.	0.75
39. Caregiver provides toys that challenge child to develop new skills.	0.80
**Variety**	**0.72**
40. Caregiver reads stories to child at least three times weekly.	0.71
41. Child eats at least one meal with caregiver and/or other children.	0.84
42. Caregiver and child visit or receive from neighbors or friends once month or so.	0.79
43. Child has three or more books of his/her own.	0.65
**Total**	**0.79**

**Table A2:** Questions for parental warmth.

Question	Cronbach’s Alpha
1. How often do you express affection by hugging, kissing, and holding this child?	0.77
2. How often do you hug or hold this child for no particular reason?	0.76
3. How often do you tell this child how happy he/she makes you?	0.80
4. How often do you have warm, close times together with this child?	0.75
5. How often do you enjoy doing things with this child?	0.79
6. How often do you feel close to this child both when he/she is happy and when he/she is upset?	0.77
**Total**	**0.80**

**Table A3:** Descriptive statistics.

Variable	Definition	Full Sample	Control Group	Treated Group	Wald Test
**Dependent variable**					
*cog*	cognitive score	94.54 (16.23)	97.73 (15.30)	92.84 (16.47)	3.05 *** (0.003)
*lang*	language score	89.78 (13.70)	93.14 (13.05)	87.99 (13.72)	3.83 *** (0.000)
*motor*	motor score	95.40 (15.75)	96.60 (14.76)	94.77 (16.24)	1.16 (0.24)
*soemo*	social-emotional score	85.41 (11.87)	87.05 (11.57)	84.53 (11.95)	2.13 ** (0.03)
**Independent variable**					
*sibling*	dummy, 1 = have siblings, 0 = no siblings	0.65 (0.47)			
*num_sibling*	sibling number	1.15 (1.20)	0.00 (0.00)	1.76 (1.06)	
**Control variable**					
*male*	dummy, 1 = male, 0 = female	0.57 (0.50)	0.62 (0.49)	0.55 (0.50)	1.32 (0.19)
*month*	month age	14.61 (5.48)	14.27 (5.70)	14.79 (5.36)	−0.95 (0.34)
*birthweight*	unit: gram	3226 (670.2)	3197 (586.1)	3242 (711.4)	−0.66 (0.51)
*birthheight*	unit: centimeter	47.96 (6.01)	48.03 (5.69)	47.92 (6.18)	0.19 (0.85)
*mage*	mother’s age	24.64 (4.98)	23.70 (5.05)	25.13 (4.88)	−2.91 *** (0.004)
*medu*	mother’s year of schooling	7.48 (3.30)	8.46 (2.97)	6.96 (3.35)	4.66 *** (0.000)
*mwork*	dummy, 1 = mother is employed, 0 = unemployed	0.55 (0.50)	0.55 (0.50)	0.54 (0.50)	0.21 (0.83)
*mminority*	dummy, 1 = mother is a minority, 0 = is not	0.25 (0.43)	0.23 (0.42)	0.27 (0.44)	−0.88 (0.38)
*momcare*	dummy, 1 = mother is the primary caregiver, 0 = is not	0.50 (0.50)	0.46 (0.50)	0.52 (0.50)	−1.20 (0.23)
*fage*	father’s age	28.63 (6.06)	26.97 (5.79)	29.51 (6.02)	−4.30 *** (0.000)
*fedu*	father’s year of schooling	7.85 (3.24)	8.90 (3.29)	7.29 (3.07)	5.10 *** (0.000)
*fwork*	dummy, 1 = father is employed, 0 = unemployed	0.84 (0.36)	0.88 (0.32)	0.82 (0.38)	1.72 * (0.09)
*fminority*	dummy, 1 = father is a minority, 0 = is not	0.23 (0.42)	0.20 (0.40)	0.24 (0.43)	−0.88 (0.38)
10,000 < *income* (yuan) ≤ 25,000	family total income in last 12 moths	0.21 (0.41)	0.20 (0.40)	0.21 (0.41)	−0.22 (0.82)
25,000 < *income* (yuan) ≤ 50,000		0.29 (0.45)	0.25 (0.43)	0.31 (0.46)	−1.48 (0.14)
50,000 < *income* (yuan) ≤ 100,000		0.24 (0.43)	0.25 (0.43)	0.24 (0.43)	0.13 (0.90)
100,000 < *income* (yuan) ≤ 250,000		0.06 (0.23)	0.08 (0.28)	0.04 (0.20)	1.88 * (0.06)
*income* (yuan) > 250,000		0.01 (0.11)	0.01 (0.11)	0.01 (0.10)	0.25 (0.80)
**Intermediate variable**					
*home*		25.50 (5.37)	26.38 (5.20)	25.04 (5.4)	2.51 ** (0.01)
*warmth*		20.51 (3.64)	21.16 (3.58)	20.17 (3.64)	2.75 *** (0.006)
observations		444	154	290	444

Note: (i) The village fixed effect is controlled by 8 village dummies, which are not presented in Table A3 because the space is limited. (ii) The statistics reported in the table are the sample mean, and the standard deviation is presented in parentheses. (iii) The Wald test reports the *t* statistics of the differences, mean (control group)—mean (treated group), and the *p* value is presented in square brackets. *, **, and *** denote *p* < 0.1, *p* < 0.05, *p* < 0.01 in two-tailed tests, respectively.

**Table A4:** The estimates for sibling effects on infants’ cognitive score.

Cognitive Score	OLS	OLS	OLS	OLS	PSM	MS
	(1)	(2)	(3)	(4)	(5)	(6)
*sibling*	−4.88 *** (1.60)	−4.43 *** (1.28)	−3.22 ** (1.42)	−3.91 ** (1.67)	−5.81 *** (2.11)	−6.46 *** (2.15)
*male*		2.58 (1.58)	2.79 * (1.55)	2.47 (1.60)		
*month*		−0.60 *** (0.13)	−0.50 *** (0.14)	−0.52 *** (0.15)		
*birthweight*		0.001 (0.001)	0.001 (0.001)	0.001 (0.001)		
*birthheight*		0.28 ** (0.11)	0.25 ** (0.12)	0.19 (0.12)		
*mage*			0.11 (0.24)	0.10 (0.21)		
*medu*			0.09 (0.30)	0.07 (0.29)		
*mwork*			−0.81 (1.87)	−1.04 (1.83)		
*mminority*			0.77 (1.83)	0.77 (1.51)		
*momcare*			1.43 (2.11)	1.36 (1.94)		
*fage*			−0.28 * (0.15)	−0.24 (0.15)		
*fedu*			0.62 ** (0.29)	0.78 ** (0.29)		
*fwork*			−0.64 (2.05)	−0.86 (2.03)		
*fminority*			1.58 (2.96)	1.50 (2.98)		
10,000 < *income* (yuan) ≤ 25,000			2.45 (2.81)	1.61 (2.59)		
25,000 < *income* (yuan) ≤ 50,000			4.30 * (2.50)	3.60 * (2.11)		
50,000 < *income* (yuan) ≤ 100,000			4.72 * (2.49)	3.87 * (2.28)		
100,000 < *income* (yuan) ≤ 250,000			1.99 (4.13)	1.25 (3.95)		
*income* (yuan) > 250,000			−2.14 (7.08)	−1.43 (8.41)		
village fixed effect	No	No	No	Yes		
Constant	97.50 *** (1.35)	86.85 *** (6.57)	83.24 *** (7.73)	87.40 *** (7.85)		
R^2^	0.0186	0.0827	0.1344	0.1573		
Observation	444	444	444	444	432	432

Note: (i) Coefficients and standard errors are reported to the nearest 0.01. (ii) In the OLS estimate, the robust standard errors clustered at the village level are presented in parentheses. In the PSM and MS estimate, ATT is reported, and the robust standard errors are obtained by the bootstrapped method with 50 replications. (iii) *, **, and *** denote *p* < 0.1, *p* < 0.05, *p* < 0.01 in two-tailed tests, respectively. (iv) The village fixed effect is controlled by 8 village dummies.

**Table A5:** The estimates for sibling effects on infants’ language score.

Language Score	OLS	OLS	OLS	OLS	PSM	MS
	(1)	(2)	(3)	(4)	(5)	(6)
*sibling*	−5.15 *** (1.35)	−5.06 *** (1.08)	−3.99 *** (1.09)	−4.22 *** (1.32)	−4.97 ** (2.01)	−5.94 *** (1.83)
*male*		−1.84 (1.40)	−1.64 (1.34)	−1.68 (1.33)		
*month*		−0.41 *** (0.14)	−0.31 ** (0.15)	−0.34 ** (0.16)		
*birthweight*		0.0004 (0.001)	0.0002 (0.001)	0.0002 (0.001)		
*birthheight*		0.18 * (0.11)	0.18 * (0.10)	0.21 ** (0.09)		
*mage*			0.15 (0.16)	0.17 (0.16)		
*medu*			0.03 (0.32)	0.02 (0.31)		
*mwork*			−1.91 (1.50)	−2.07 (1.50)		
*mminority*			−1.25 (1.90)	−1.46 (1.75)		
*momcare*			2.08 (1.54)	2.10 (1.46)		
*fage*			−0.26 (0.16)	−0.25 (0.16)		
*fedu*			0.51 * (0.27)	0.58 ** (0.27)		
*fwork*			−1.65 (1.75)	−1.62 (1.81)		
*fminority*			4.46 ** (2.02)	3.80 * (1.91)		
10,000 < *income* (yuan) ≤ 25,000			0.55 (2.37)	0.31 (2.27)		
25,000 < *income* (yuan) ≤ 50,000			0.77 (1.97)	0.79 (1.77)		
50,000 < *income* (yuan) ≤ 100,000			0.73 (1.99)	0.62 (1.77)		
100,000 < *income* (yuan) ≤ 250,000			5.00 (3.69)	5.12 (3.85)		
*income* (yuan) > 250,000			−7.10 (5.01)	−6.41 (5.97)		
village fixed effect	No	No	No	Yes		
Constant	92.89 *** (1.01)	90.30 *** (5.24)	88.47 *** (6.94)	89.86 *** (6.26)		
R^2^	0.0286	0.0709	0.1415	0.1578		
Observation	444	444	444	444	432	432

Note: (i) Coefficients and standard errors are reported to the nearest 0.01. (ii) In the OLS estimate, the robust standard errors clustered at the village level are presented in parentheses. In the PSM and MS estimate, ATT is reported, and the robust standard errors are obtained by the bootstrapped method with 50 replications. (iii) *, **, and *** denote *p* < 0.1, *p* < 0.05, *p* < 0.01 in two-tailed tests, respectively. (iv) The village fixed effect is controlled by 8 village dummies.

**Table A6:** The estimates for sibling effects on infants’ motor score.

Motor Score	OLS	OLS	OLS	OLS	PSM	MS
	(1)	(2)	(3)	(4)	(5)	(6)
*sibling*	−1.83 (1.57)	−2.35 * (1.25)	−1.32 (1.37)	−2.17 (1.44)	−3.14 (2.27)	−3.58 (2.09)
*male*		0.47 (1.72)	0.86 (1.73)	0.84 (1.76)		
*month*		0.87 *** (0.12)	0.94 *** (0.13)	0.88 *** (0.14)		
*birthweight*		0.002 * (0.001)	0.002 * (0.001)	0.002 * (0.001)		
*birthheight*		0.01 (0.12)	0.02 (0.13)	0.02 (0.12)		
*mage*			0.10 (0.16)	0.08 (0.18)		
*medu*			0.17 (0.26)	0.15 (0.26)		
*mwork*			0.05 (1.97)	0.08 (1.50)		
*mminority*			−1.96 (2.02)	−2.38 (1.79)		
*momcare*			0.33 (1.93)	0.62 (1.70)		
*fage*			−0.27 (0.17)	−0.21 (0.17)		
*fedu*			0.43 * (0.26)	0.54 * (0.27)		
*fwork*			−5.45 *** (1.89)	−5.75 *** (1.84)		
*fminority*			2.69 (2.81)	1.01 (2.62)		
10,000 < *income* (yuan) ≤ 25,000			2.32 (2.68)	1.74 (2.54)		
25,000 < *income* (yuan) ≤ 50,000			4.03 * (2.36)	3.71 * (2.05)		
50,000 < *income* (yuan) ≤ 100,000			3.36 (2.52)	2.61 (2.25)		
100,000 < *income* (yuan) ≤ 250,000			4.94 (5.03)	4.38 (4.59)		
*income* (yuan) > 250,000			−6.53 (9.40)	−4.63 (10.75)		
village fixed effect	No	No	No	Yes		
Constant	96.45 *** (1.03)	76.38 *** (5.69)	78.97 *** (7.71)	82.94 *** (7.71)		
R^2^	0.0026	0.1088	0.1639	0.1995		
Observation	444	444	444	444	432	432

Note: (i) Coefficients and standard errors are reported to the nearest 0.01. (ii) In the OLS estimate, the robust standard errors clustered at the village level are presented in parentheses. In the PSM and MS estimate, ATT is reported, and the robust standard errors are obtained by the bootstrapped method with 50 replications. (iii) *, **, and *** denote *p* < 0.1, *p* < 0.05, *p* < 0.01 in two-tailed tests, respectively. (iv) The village fixed effect is controlled by 8 village dummies.

**Table A7:** The estimates for sibling effects on infants’ social-emotional score.

Social-Emotional Score	OLS	OLS	OLS	OLS	PSM	MS
	(1)	(2)	(3)	(4)	(5)	(6)
*sibling*	−2.51 ** (1.17)	−2.63 *** (0.97)	−2.19 ** (0.91)	−2.14 ** (0.85)	−4.03 ** (1.75)	−3.48 ** (1.41)
*male*		−0.50 (1.33)	−0.38 (1.37)	−0.65 (1.35)		
*month*		0.18 * (0.10)	0.20 * (0.10)	0.21 ** (0.09)		
*birthweight*		0.0001 (0.0008)	0.0001 (0.0008)	0.0002 (0.0008)		
*birthheight*		0.12 (0.14)	0.12 (0.13)	0.11 (0.11)		
*mage*			−0.21 (0.21)	−0.22 (0.20)		
*medu*			0.23 (0.27)	0.21 (0.27)		
*mwork*			2.57 * (1.41)	1.89 (1.47)		
*mminority*			−0.07 (1.77)	−0.03 (1.84)		
*momcare*			0.58 (1.59)	0.80 (1.65)		
*fage*			0.08 (0.19)	0.11 (0.18)		
*fedu*			0.11 (0.24)	0.12 (0.24)		
*fwork*			−1.53 (1.83)	−1.43 (1.77)		
*fminority*			2.61 (2.04)	1.35 (2.10)		
10,000 < *income* (yuan) ≤ 25,000			0.77 (2.25)	0.37 (2.53)		
25,000 < *income* (yuan) ≤ 50,000			−0.30 (2.01)	−0.38 (2.00)		
50,000 < *income* (yuan) ≤ 100,000			−0.67 (1.96)	−0.69 (1.92)		
100,000 < *income* (yuan) ≤ 250,000			0.41 (2.87)	0.40 (3.08)		
*income* (yuan) > 250,000			−0.62 (2.91)	−0.79 (2.71)		
village fixed effect	No	No	No	Yes		
Constant	86.79 *** (1.03)	78.56 *** (6.93)	77.20 *** (7.58)	80.23 *** (6.87)		
R^2^	0.0082	0.0215	0.0498	0.0788		
Observation	444	444	444	444	432	432

Note: (i) Coefficients and standard errors are reported to the nearest 0.01. (ii) In the OLS estimate, the robust standard errors clustered at the village level are presented in parentheses. In the PSM and MS estimate, ATT is reported, and the robust standard errors are obtained by the bootstrapped method with 50 replications. (iii) *, **, and *** denote *p* < 0.1, *p* < 0.05, *p* < 0.01 in two-tailed tests, respectively. (iv) The village fixed effect is controlled by 8 village dummies.

**Table A8:** The OLS estimates for sibling number effect on infants’ developmental skills.

Bayley Score	*Cog*	*Lang*	*Motor*	*Soemo*
	(1)	(2)	(3)	(4)
*num_sibling*	−1.66 *** (0.58)	−1.52 ** (0.69)	−0.15 (0.72)	−0.85 * (0.43)
infant characteristics	Yes	Yes	Yes	Yes
family characteristics	Yes	Yes	Yes	Yes
village fixed effect	Yes	Yes	Yes	Yes
R^2^	0.1581	0.1537	0.1960	0.0784
Observation	444	444	444	444

Note: (i) Coefficients and standard errors are reported to the nearest 0.01. (ii) The robust standard errors clustered at the village level are presented in parentheses. (iii) *, **, and *** denote *p* < 0.1, *p* < 0.05, *p* < 0.01 in two-tailed tests, respectively.

**Table A9:** The estimates for the mediation effect of home environment: robustness check.

**Bayley Score**	***Cog***	***Lang***	***Motor***	***Soem**o*
*num_sibling*	−2.03 *** (0.64)	−2.03 *** (0.54)	−0.65 (0.62)	−1.05 ** (0.47)
**Intermediate variable**	***home***	***home***	***home***	***home***
*num_sibling*	−0.47 ** (0.21)	−0.47 ** (0.21)	−0.47 ** (0.21)	−0.47 ** (0.21)
**Bayley score**	***cog***	***lang***	***motor***	***soemo***
*num_sibling*	−1.88 *** (0.64)	−1.78 *** (0.53)	−0.47 (0.62)	−0.95 ** (0.47)
*home*	0.32 ** (0.14)	0.51 *** (0.12)	0.39 *** (0.14)	0.19 * (0.10)
Sobel test	−0.15 (0.10)	−0.24 ** (0.12)	\	−0.09 (0.06)
bootstrap test	−0.15 * (0.09)	−0.2433 ** (0.1196)	\	−0.09 (0.07)
percentile confidence interval of indirect effect	(−0.35, −0.01)	(−0.49, −0.02)	\	(−0.24, 0.01)
bias-corrected confidence interval of indirect effect	(−0.39, −0.03)	(−0.50, −0.03)	\	(−0.26, 0.004)
mediated proportion of total effect	7.4%	12.0%	\	8.7%

Note: (i) Coefficients and standard errors are reported to the nearest 0.01. (ii) The robust standard errors clustered at the village level are presented in parentheses. (iii) *, **, and *** denote *p* < 0.1, *p* < 0.05, *p* < 0.01 in two-tailed tests, respectively. (iv) The bootstrap test is based on resampling with 1000 replications; a 95% confidence interval is reported.

**Table A10:** The estimates for the mediation effect of parental warmth: robustness check.

**Bayley Score**	***Cog***	***Lang***	***Motor***	***Soemo***
*num_sibling*	−2.03 *** (0.64)	−2.03 *** (0.54)	−0.65 (0.62)	−1.05 ** (0.47)
**Intermediate variable**	***warmth***	***warmth***	***warmth***	***warmth***
*num_sibling*	−0.32 ** (0.14)	−0.32 ** (0.14)	−0.32 ** (0.14)	−0.32 ** (0.14)
**Bayley score**	***cog***	***lang***	***motor***	***soemo***
*num_sibling*	−1.98 *** (0.64)	−1.91 *** (0.54)	−0.64 (0.63)	−0.90 * (0.47)
*warmth*	0.14 (0.21)	0.37 ** (0.18)	0.03 (0.21)	0.44 *** (0.15)
Sobel test	−0.05 (0.07)	−0.12 (0.08)	\	−0.14 * (0.08)
bootstrap test	−0.05 (0.08)	−0.12 (0.09)	\	−0.14 * (0.09)
percentile confidence interval of indirect effect	(−0.25, 0.10)	(−0.33, 0.01)	\	(−0.37, 0.02)
bias-corrected confidence interval of indirect effect	(−0.30, 0.06)	(−0.35, 0.01)	\	(−0.43, −0.004)
mediated proportion of total effect	2.3%	5.9%	\	13.7%

Note: (i) Coefficients and standard errors are reported to the nearest 0.01. (ii) The robust standard errors clustered at the village level are presented in parentheses. (iii) *, **, and *** denote *p* < 0.1, *p* < 0.05, *p* < 0.01 in two-tailed tests, respectively. (iv) The bootstrap test is based on resampling with 1000 replications; a 95% confidence interval is reported.

**Table A11:** The OLS estimates for home environment scales’ effects on cognitive score.

Cognitive Score	(1)	(2)	(3)	(4)	(5)	(6)
*respon*	0.05 (0.27)					
*accept*		0.10 ** (0.02)				
*organ*			0.09 ** (0.05)			
*learnm*				0.16 *** (0.000)		
*involv*					0.04 (0.43)	
*variety*						0.10 ** (0.02)
infant characteristics	Yes	Yes	Yes	Yes	Yes	Yes
family characteristics	Yes	Yes	Yes	Yes	Yes	Yes
village fixed effect	Yes	Yes	Yes	Yes	Yes	Yes
R^2^	0.1486	0.1478	0.1469	0.1488	0.1465	0.1463
Observation	444	444	444	444	444	444

Note: (i) Standardized coefficients and two-tailed *p*-value are reported to the nearest 0.01. (ii) Two-tailed *p*-values are presented in parentheses. (iii) *, **, and *** denote *p* < 0.1, *p* < 0.05, *p* < 0.01 in two-tailed tests, respectively.

**Table A12:** The OLS estimates for home environment scales’ effects on language score.

Language Score	(1)	(2)	(3)	(4)	(5)	(6)
*respon*	0.15 *** (0.001)					
*accept*		0.06 (0.13)				
*organ*			0.17 *** (0.000)			
*learnm*				0.16 *** (0.000)		
*involv*					0.08 * (0.09)	
*variety*						0.14 *** (0.03)
infant characteristics	Yes	Yes	Yes	Yes	Yes	Yes
family characteristics	Yes	Yes	Yes	Yes	Yes	Yes
village fixed effect	Yes	Yes	Yes	Yes	Yes	Yes
R^2^	0.1508	0.1402	0.1462	0.1450	0.1407	0.1418
Observation	444	444	444	444	444	444

Note: (i) Standardized coefficients and two-tailed *p*-value are reported to the nearest 0.01. (ii) Two-tailed *p*-values are presented in parentheses. (iii) *, **, and *** denote *p* < 0.1, *p* < 0.05, *p* < 0.01 in two-tailed tests, respectively.

**Table A13:** The OLS estimates for home environment scales’ effects on social-emotional score.

Social-Emotional Score	(1)	(2)	(3)	(4)	(5)	(6)
*respon*	0.07 (0.16)					
*accept*		0.02 (0.76)				
*organ*			0.02 (0.65)			
*learnm*				0.11 ** (0.02)		
*involv*					0.07 (0.22)	
*variety*						0.03 (0.49)
infant characteristics	Yes	Yes	Yes	Yes	Yes	Yes
family characteristics	Yes	Yes	Yes	Yes	Yes	Yes
village fixed effect	Yes	Yes	Yes	Yes	Yes	Yes
R^2^	0.0795	0.0739	0.0747	0.0808	0.0794	0.0735
Observation	444	444	444	444	444	444

Note: (i) Standardized coefficients and two-tailed *p*-value are reported to the nearest 0.01. (ii) Two-tailed *p*-values are presented in parentheses. (iii) *, **, and *** denote *p* < 0.1, *p* < 0.05, *p* < 0.01 in two-tailed tests, respectively.
